# Effect of chlorogenic acids on fatigue and sleep in healthy males: A randomized, double‐blind, placebo‐controlled, crossover study

**DOI:** 10.1002/fsn3.861

**Published:** 2018-10-19

**Authors:** Ryuji Ochiai, Kazuichi Tomonobu, Ippei Ikushima

**Affiliations:** ^1^ Biological Science Laboratories Kao Corporation Tokyo Japan; ^2^ Health Care Food Research Laboratories Kao Corporation Tokyo Japan; ^3^ SOUSEIKAI Sumida Hospital Tokyo Japan

**Keywords:** chlorogenic acids, coffee, fatigue, sleep

## Abstract

Chlorogenic acids (CGAs) are found in abundance in coffee beans and have numerous health benefits. This study investigated the effect of CGAs extracted from coffee beans on fatigue and sleep in healthy participants. This crossover study involved 16 men (aged 30–54 years) who were daytime workers with weekends off work. The participants were randomized into two groups: One group was given a placebo beverage, and the other was given an active beverage containing 300 mg of CGAs. The test beverages were consumed for 13 days starting on a Sunday. The evaluation criteria were feelings of fatigue and sleep quality, sleep‐related indices recorded by an activity meter, and biomarkers. Feelings of fatigue and sleep‐related indices during the first (first half of week 1) and second (second half of week 2) halves of the consumption periods were compared. Within the first half of week 1, no differences in feelings of fatigue were observed between the groups. However, during the second half of week 2, fatigue upon awakening and sleep quality in the active group were significantly improved compared with those in the placebo group. Sleep efficiency and total nocturnal awakening time were significantly deteriorated in the second half of week 2 compared with the first half of week 1 in the placebo group. Furthermore, significant differences in these indices were observed between the two groups in the second half of week 2. These results suggest that the daily consumption of CGA‐containing beverages may improve fatigue upon awakening and sleep quality.

## 1 INTRODUCTION

Fatigue, the most common type of physical discomfort experienced by people worldwide (David et al., 1990), is both a perceptual problem and a substantial social problem in terms of the productivity loss caused by decreased work efficiency among tired workers (Yamazaki et al., 2007). Moreover, fatigue decreases the quality of life (QOL) of many people (Jelsness‐Jørgensen, Bernklev, Henriksen, Torp, & Moum, 2011). Major causes of fatigue are mental/physical stress, disrupted sleep, and short sleep times resulting in drowsiness, which are common symptoms of an underlying pathological disorder (Ancoli‐Israel, Moore, & Jones, 2001). In modern society, reduced quality of sleep is thought to correlate with factors in everyday life, such as high stress levels, lack of physical exercise, and problems with sleeping environments (Doi, Minowa, & Tango, 2003); factors affecting sleep quality lead to the accumulation of fatigue, causing problems in day‐to‐day activities.

Interestingly, the research unit of the Ministry of Health, Labour and Welfare reported that 60% of the Japanese population had feelings of fatigue, and 37% experienced chronic fatigue that lasted more than 6 months. Those with chronic fatigue experienced characteristic symptoms of busyness, stress, difficulty of falling asleep, and interrupted sleep or early‐morning awakening more frequently than those not experiencing fatigue (Watanabe & Kuratsune, 2006). Symptoms of fatigue are reversed by rest in normal cases, and sleep thus plays an important role in fatigue recovery (Dawson & McCulloch, 2005). Therefore, evaluating sleep when conducting research on fatigue is important.

Recently, we showed, for the first time, the effect of continuously consuming chlorogenic acids (CGAs) extracted from coffee beans on shortened sleep latency (Park et al., 2017). Coffee beans contain polyphenols, which have antioxidant properties, and CGAs are the major polyphenol constituents (Clifford, 1999). Many studies have reported the health benefits of coffee, especially regarding its protective effects against diseases, such as type II diabetes, hypertension, Alzheimer's disease, and Parkinson's disease (Cano‐Marquina, Tarín, & Cano, 2013; van Dam & Feskens, 2002; Eskelinen, Ngandu, Tuomilehto, Soininen, & Kivipelto, 2009; Hernán, Takkouche, Caamaño‐Isorna, & Gestal‐Otero, 2002; Jee, He, Whelton, Suh, & Klag, 1999). However, since roasted coffee normally contains caffeine, it was reported to deteriorate rather than improve sleep (Clark & Landolt, 2017). The observed health benefits are at least partially due to the effect of CGAs found in coffee (Butt & Sultan, 2011; Ranheim & Halvorsen, 2005). Recent studies on humans have shown that lipid oxidation and improvements in insulin resistance, vascular endothelial function, and brain function are all due to the consumption of CGAs (300‐600 mg, equivalent to quantities contained in 1~2 cups of coffee) (Kato, Ochiai, Kozuma, Sato, & Katsuragi, 2018; Lecoultre et al., 2014; Ochiai, Sugiura, Otsuka, Katsuragi, & Hashiguchi, 2015; Soga, Ota, & Shimotoyodome, 2013), demonstrating that CGAs exert a wide range of benefits. Herein, we investigated the effects of CGA consumption on fatigue improvement and sleep in healthy participants who experienced increased levels of fatigue toward the weekend.

## 2 PARTICIPANTS AND METHODS

### 2.1 Participants

A total of 16 healthy Japanese men aged 30–54 years (mean age, 42.9) were recruited for this study. Participants were daytime workers who had the weekends off and felt fatigued upon awakening during the weekdays but recovered from fatigue over the weekend. Their main job descriptions did not involve physical activity. Data on daytime fatigue were collected using questionnaires and the Epworth Sleepiness Scale (ESS) (Takegami et al., 2009). Those who were currently receiving drugs or medical treatment, had a past medical history of cardiovascular disease or seizures due to neurological disorders, had food allergies, smoked, consumed more than 30 g/day of alcohol, could not comply with the daily life restrictions, or were considered inappropriate for enrollment by the principal investigator were excluded from this study. Participants were recruited by TES Holdings Co., Ltd. (Tokyo, Japan).

### 2.2 Study procedures

The study protocol was approved by the ethics committee of the Bokushinkai Medical Corporation Shirogane EXE Clinic (approval number: 2013‐0501; approval date: 8 May 2013). All participants were provided with written explanations of the scope, features, aims, and potential risks associated with this study by the principal investigator and signed written consent. This study was conducted in accordance with the Declaration of Helsinki under careful supervision by the principal investigator and directed entirely by TES Holdings Co., Ltd. from May 2013 to July 2013 at Medical Co. LTA Sumida Hospital (Tokyo, Japan).

### 2.3 Materials

The test beverages consisted of an active beverage containing 300 mg of CGAs in 100 ml of water and an equal volume of a placebo beverage containing no CGAs in the same type of bottle and with the same taste as the active beverage. The test beverages were prepared specifically for this study, and both beverages had an energy content of less than 10 kcal. CGAs were extracted from *Coffea canephora* green coffee beans using a hot water extraction method and decaffeinated using activated carbon; the dry powder was obtained by spray drying (Ochiai et al., 2014). The caffeine level was adjusted to a small amount (< 3 mg/100 ml) because caffeine impairs sleep function (Clark & Landolt, 2017). The composition of the CGAs consisted of three types of quinic acid derivatives: 67.2% caffeoylquinic acids, 13.8% feruloylquinic acids, and 18.7% dicaffeoylquinic acids.

### 2.4 Study protocol

This study was designed as a randomized, double‐blind, placebo‐controlled, crossover study. Each crossover phase was 5 weeks long and comprised 2 weeks of beverage consumption repeated twice with a 1‐week washout period in between. Measurements were taken at the beginning and end of each consumption period (hereafter called “pre” and “post”) for a total of four times. Pathological evaluations and plasma/urine tests were conducted prior to randomization, and these initial data were used by the investigator to select participants for enrollment based on the inclusion and exclusion criteria. Selected participants were then randomized to either the active or placebo beverage groups. The consumption of either beverage was started on a Sunday and continued to the second Friday for 13 consecutive days. After a 1‐week washout, the other test beverage was consumed for an additional 13 days. The test beverages were recommended to be consumed at least 30 min prior to going to bed. The participants were recruited to the hospital at 10:00 a.m. on the Saturday before the study initiation (pre) and on the Saturday after completion (post) for pathological and hematological examinations. Alcohol was prohibited after dinner the day before the measurements. Similarly, the participants were asked to collect their total overnight urine in the morning after awakening and bring it to the hospital for examination.

During the study, the participants were asked to make subjective evaluations of fatigue feelings and sleep quality using the visual analog scale (VAS) before going to bed and after awakening every day. Sleep‐related indices were analyzed over the first 3 days (Sunday, Monday, and Tuesday) and over the 3 days prior to completion (Tuesday, Wednesday, and Thursday).

Throughout the study period, the participants were asked to maintain their normal lifestyle but to refrain from consuming caffeine or alcohol‐containing drinks after dinner, as these substances may affect sleep. Personal computers, smartphones, and TV games were prohibited during the hour prior to bedtime.

### 2.5 Measurements

#### 2.5.1 Subjective evaluation

Feelings of fatigue and sleep quality were evaluated using a 100‐mm horizontal line VAS (Lee, Hicks, & Nino‐Murcia, 1991). The leftmost score at 0 mm was deemed the “best” or “good sleep” state, and the rightmost score at 100 mm was deemed the “worst” or “couldn't sleep” state. Evaluations were made every day after awakening and before going to bed. For daytime fatigue assessed in the prerandomization test, daytime sleepiness was evaluated using the Japanese version of the ESS, which is a self‐administered questionnaire consisting of eight categories, each with a question related to sleep and drowsiness during daily activities. Each question is answered using a four‐point scale (0–3), and a total ESS score >10 indicates pathological sleepiness (Takegami et al., 2009).

#### 2.5.2 Sleep‐related indices

Activity levels during sleep were measured over the first 3 days (Sunday, Monday, and Tuesday) and over the 3 days prior to completion (Tuesday, Wednesday, and Thursday) using a wearable physiological sensor (NEM‐T1, Toshiba, Japan), which functioned as an activity meter. The participants wore this device on their nondominant wrist and switched it on before going to bed; the device was then switched off and removed in the morning after awakening. The wearable physiological sensor was 55 × 57 × 14 mm in size, weighed 40 g, and operated on a rechargeable battery. Like in actigram analysis, the activity levels during sleep were analyzed using a three‐axis accelerometer, where values greater than 0.1 G were analyzed with Cole's algorithm (Cole, Kripke, Gruen, Mullaney, & Gillin, 1992; Inano, Mizumori, Kobayashi, Sumiya, & Yatani, 2013; Kamata et al., 2011).

#### 2.5.3 Physical and circulatory evaluation

The height, weight, systolic blood pressure, diastolic blood pressure, pulse, and body temperature of each participant were measured before and after consumption of the test beverages (“pre” and “post”). Height was measured only once prior to randomization.

#### 2.5.4 Plasma/urine measurements

Plasma and urine were sampled in the prerandomization test and during pre‐ and post‐morning fasting. Plasma amino acids, serum cortisol, and dehydroepiandrosterone sulfate (DHEA‐S) were detected and analyzed at the Health Sciences Research Institute, Inc. in Kanagawa, Japan. For the urine samples, the specific gravity, pH, ketone bodies, occult blood reaction, urobilinogen, bilirubin, qualitative protein, and qualitative glucose were measured at prerandomization. For the pre‐ and postmeasurements, 8‐OHdG, isoprostane, and creatinine in the urine samples collected overnight were analyzed at the Japan Institute for the Control of Aging (Shizuoka, Japan).

### 2.6 Statistical analysis

The Mann–Whitney *U* test was used to compare VAS scores as well as the measurements of subjective fatigue and sleep perceptions between the two groups. To analyze sleep‐related indices, the values on Monday and Tuesday of week 1 (first half of week 1) and on Wednesday and Thursday of week 2 (second half of week 2) were used to make comparisons within and between groups using a linear mixed model and the Bonferroni post hoc test. For plasma and urine tests, comparisons within and between the pre and post groups were made using initial values as the covariates in the linear mixed model and the Bonferroni post hoc test. Student's *t* test was used to compare the consumption rates of the test beverages between the groups during the study period. We considered a *p* value of <0.05 to be significant. Statistics are presented as the mean ± standard deviation. SPSS statistics 19 (SPSS Japan Inc., Tokyo) was used for statistical analysis.

## 3 RESULTS

### 3.1 Characteristics of the participants

All 16 participants completed the study, and their characteristics are shown in Table 1. One participant experienced short hours of sleep due to work‐related problems and was excluded from the analysis. No specific adverse events were reported during this study. The consumption rate of the test beverages was 100%.

**Table 1:** Characteristics of the subjects

	Mean ± *SD*	Range
Number	16 (Male)	
Age (y)	42.9 ± 7.1	(30–54)
Weight (kg)	68.1 ± 9.6	(44.7–83.4)
BMI (kg/m^2^)	22.8 ± 2.5	(17.2–28.0)
Body fat (%)	16.8 ± 4.9	(13.0–25.4)
Epworth sleepiness scale score	13.3 ± 1.3	(11–15)

*Note*

BMI, body mass index.

### 3.2 Subjective evaluation

**Figure 1:**
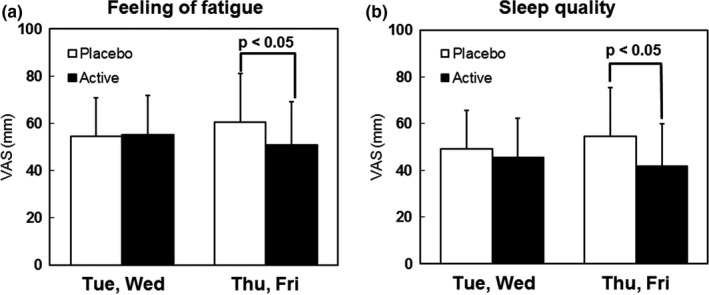
Subjective evaluation on awakening: (a) Feeling of fatigue; (b) Sleep quality (good sleep). Values are expressed as mean ± *SD* (*N* = 15). 1) Average of 2 days’ (Tuesday and Wednesday) data on awakening in the week 1 during the test period. 2) Average of 2 days’ (Thursday and Friday) data on awakening in the week 2 during the test period. *p* value: significant difference between Placebo and Active beverage (Mann‐Whitney *U* test)

The VAS evaluations of fatigue upon awakening and sleep quality are shown in Figure 1. For the VAS scores, the mean value for Tuesday and Wednesday of the first week (first half of week 1) was compared to that for Thursday and Friday of the second week (second half of week 2). There were no differences between the active and placebo groups in the first half of week 1; however, feelings of fatigue and sleep quality were both significantly better among the active group in the second half of week 2.

### 3.3 Sleep‐related indices

For the sleep‐related indices shown in Table 2, the mean value for Monday and Tuesday of the first week (first half of week 1) was compared to that for Wednesday and Thursday of the second week (second half of week 2). There were no differences in total sleep time, sleep latency, or frequency of nocturnal awakening between the active and placebo groups. In the placebo group, the sleep efficiency was decreased significantly in the second half of week 2 compared with that in the first half of week 1, whereas the total nocturnal awakening time was significantly increased. Significant differences in these two indices were observed between the two groups in the second half of week 2.

**Table 2:** Sleep‐related indices

	Beverage	First half of week 1	Second half of week 2	*P1*	*P2*
Total sleep time (min)	Placebo	352 ± 65	358 ± 46	0.747	0.699
	Active	365 ± 54	363 ± 64	0.907	
Sleep efficiency (%)	Placebo	96.2 ± 3.9	92.3 ± 11.0	0.015	0.046
	Active	96.5 ± 4.1	95.4 ± 3.1	0.603	
Sleep latency (min)	Placebo	5.7 ± 2.9	5.6 ± 3.0	0.969	0.705
	Active	5.4 ± 2.7	5.9 ± 2.4	0.556	
Total nocturnal awakening time (min)	Placebo	13.6 ± 14.4	29.6 ± 45.0	0.013	0.039
	Active	13.3 ± 16.5	17.0 ± 12.4	0.669	
Frequency of nocturnal awakenings (number)	Placebo	5.1 ± 3.2	6.3 ± 6.8	0.111	0.706
	Active	5.1 ± 4.4	6.1 ± 4.1	0.277	

*Notes*

Values are expressed as the mean ± *SD* (*N* = 15).

First half of week 1: Average of 2 days’ (Monday and Tuesday) data in week 1 during the test period.

Second half of week 2: Average of 2 days’ (Wednesday and Thursday) data in week 2 during the test period.

P1 value; significant difference between the first half of week 1 and the second half of week 2 (Bonferroni post hoc test).

P2 value; significant difference between the placebo and active beverage in the second half of week 2 (Bonferroni post hoc test).

### 3.4 Plasma and urinary markers

The plasma and urinary stress markers analyzed herein are shown in Table 3. In the active group, the plasma DHEA‐S and DHEA‐S/cortisol levels were significantly increased in the “post” group compared to those in the “pre” group. There were no significant differences in the other markers between the two groups. The plasma amino acid levels are shown in Table 4. In both the active and placebo groups, cystine was significantly increased in the “post” group compared with that in the “pre” group. In the active group, the levels of citrulline and ornithine were significantly increased in the “post” group compared to that in the “pre” group, whereas the levels of arginine were significantly decreased. A significant difference was observed in the ornithine and arginine levels between the active and placebo groups.

**Table 3:** Biochemical markers

	Placebo		*P1*	Active		*P1*
	Pre	Post		Pre	Post	
Cortisol (ug/dl)^a^	10.1 ± 3.4	10.1 ± 3.7	0.946	10.4 ± 3.9	9.2 ± 3.0	0.188
DHEA‐S (ug/dl)^a^	229 ± 94	239 ± 98	0.263	233 ± 88	250 ± 89	0.048
DHEA‐S/cortisol^a^	26 ± 16	27 ± 14	0.726	26 ± 13	30 ± 13	0.032
8‐OHdG/Cre (ng/mg Cre)^b^	7.1 ± 1.7	7.1 ± 2.3	0.976	7.2 ± 2.0	6.8 ± 1.4	0.298
Isoprostane/Cre (ng/mg Cre)^b^	3.1 ± 2.6	2.2 ± 0.9	0.067	2.4 ± 1.0	2.1 ± 1.0	0.476

*Notes*

P1 value; significant difference between “pre” and “post” (Bonferroni post hoc test).

^a^Plasma; ^b^Urinary.

**Table 4:** Plasma amino acids

	Placebo		*P1*	Active		*P1*	*P2*
	Pre	Post		Pre	Post		
Alanine	364 ± 101	368 ± 83	0.797	387 ± 86	358 ± 66	0.075	0.148
Asparagine	43 ± 10	45 ± 7	0.107	46 ± 6	45 ± 7	0.378	0.080
Cystine	41 ± 5	46 ± 7	0.006	42 ± 7	46 ± 6	0.004	0.922
Glutamic acid	36 ± 14	41 ± 15	0.025	39 ± 14	44 ± 15	0.052	0.821
Phenylalanine	53 ± 9	54 ± 7	0.596	55 ± 8	54 ± 6	0.623	0.470
Glycine	212 ± 62	221 ± 46	0.243	224 ± 39	216 ± 43	0.274	0.112
Histidine	77 ± 10	77 ± 6	0.974	78 ± 10	77 ± 8	0.534	0.676
Isoleucine	64 ± 18	64 ± 12	0.862	67 ± 15	64 ± 12	0.270	0.509
Lysine	176 ± 38	180 ± 31	0.596	184 ± 30	181 ± 33	0.638	0.480
Leucine	127 ± 24	130 ± 20	0.670	129 ± 22	128 ± 22	0.826	0.648
Methionine	25 ± 8	24 ± 5	0.820	26 ± 4	25 ± 4	0.160	0.400
Proline	163 ± 52	156 ± 36	0.490	155 ± 93	149 ± 49	0.572	0.929
Glutamine	493 ± 72	484 ± 45	0.558	484 ± 55	472 ± 73	0.376	0.831
Arginine	77 ± 24	80 ± 17	0.508	88 ± 16	81 ± 14	0.028	0.043
Serine	110 ± 24	114 ± 22	0.618	111 ± 19	110 ± 24	0.977	0.709
Threonine	121 ± 33	129 ± 26	0.211	129 ± 23	131 ± 23	0.674	0.553
Valine	225 ± 48	233 ± 48	0.423	233 ± 41	239 ± 34	0.586	0.855
Tryptophan	56 ± 10	57 ± 10	0.952	55 ± 7	59 ± 7	0.145	0.320
Tyrosine	56 ± 14	57 ± 12	0.676	59 ± 12	57 ± 11	0.349	0.339
Aminobutanoic acid	19 ± 3	19 ± 5	0.964	18 ± 3	20 ± 4	0.163	0.337
Citrulline	35 ± 5	37 ± 6	0.141	35 ± 4	38 ± 5	0.018	0.498
Ornithine	63 ± 16	61 ± 13	0.420	60 ± 12	66 ± 11	0.046	0.048

*Notes*

P1 value; significant difference between “pre” and “post” (Bonferroni post hoc test).

P2 value; interaction between the treatment and time effect by linear mixed‐model analysis.

## 4 DISCUSSION

This is the first study in which the daily consumption of CGAs was shown to simultaneously improve fatigue and sleep quality in human participants. Participants recruited for this study were daytime workers who experienced fatigue during the weekdays as the weekend approached, and the effect of CGAs on suppressing fatigue over the weekend was evaluated. During the study, fatigue upon awakening tended to increase in the second half of week 2 in comparison with that during the first half of week 1 in the placebo group; however, this increase in fatigue was significantly suppressed in the active group. We will first discuss the effect of this active beverage in relation to sleep and plasma biomarkers.

First, in terms of sleep, the mean ESS score of the participants reached 13.3, which was similar to values reported by participants who experienced excessive daytime sleepiness (Ohayon, 2008). The mean total sleep time of the participants was approximately 6 h during the study period, which was similar to the length of sleep of an average Japanese person (Ministry of Health, Labour and Welfare, Japan, 2011). However, sleep efficiency decreased and total nocturnal awakening time increased significantly in the second half of week 2 compared with those values during the first half of week 1 in the placebo group, suggesting that the quality of sleep, not the total sleep time, decreased in the second half of week 2 among these participants. It is thought that sleep deprivation and arousal are induced by psychological stress, but no research results have verified this hypothesis (Nagai, Hoshide, & Kario, 2010). The results of the current study suggest that the quality of sleep in the second half of week 2 decreased due to the accumulation of psychological stress in daily work. In contrast, this decrease in sleep quality was suppressed in the active group in the second half of week 2, suggesting that CGAs consumed at a dose lower than that previously reported (Park et al., 2017) may be sufficient to improve sleep quality. Park et al. reported that CGAs act in part by increasing parasympathetic nervous system activation (Park et al., 2017). Furthermore, acute psychological stress has been reported to decrease sleep quality and parasympathetic nerve activity during sleep (Hall et al., 2004). Therefore, these results suggest that CGAs suppress the effect of psychological stress on decreasing sleep quality by affecting the autonomic nervous system, thereby promoting recovery from fatigue via sleep.

Second, DHEA‐S, an adrenocortical hormone, and DHEA‐S/cortisol, a marker of psychological stress associated with the hypothalamic–pituitary–adrenal (HPA) axis (Morgan et al., 2004), were both significantly increased in only the active group. Psychological stress, such as anxiety, is known to increase HPA axis activity and decrease DHEA‐S/cortisol levels (Kim, Lee, & Ahn, 2010). Oxidative stress was recently associated with anxiety levels (Hovatta et al., 2005), and CGAs are well‐known antioxidants (Clifford, 1999); therefore, the increase in DHEA‐S/cortisol levels observed in this study may have been due to both direct and indirect CGA effects. Indirectly, our results suggested that CGAs act via improving sleep quality. Plasma amino acid analysis revealed that citrulline and ornithine were increased significantly in only the active group, with significantly higher ornithine levels being observed in this group compared to those in the placebo group. These two substances are important in the metabolism of ammonia to urea in living organisms (Wilkinson, Smeeton, & Watt, 2010), and high levels of ammonia are known to decrease energy production in the brain (Felipo & Butterworth, 2002). As citrulline and ornithine were increased due to CGA consumption, CGAs may also be beneficial in recovery from physical activity‐related fatigue.

This study did have several limitations. First, because the participants were limited to middle‐aged men who were daytime workers with weekends off, the study findings are not generalizable. Further studies must be conducted in groups with more diverse profiles to draw more general conclusions. Second, the sample size was insufficient to draw a definite conclusion. Third, a detailed mechanism describing the effect of CGAs on sleep quality improvement must be elucidated. However, our study revealed that consuming CGAs daily effectively improves sleep quality and fatigue upon awakening.

## CONFLICT OF INTEREST

This work was financially supported by Kao Corporation. Ryuji Ochiai, Kazuichi Tomonobu, and Yoshihisa Katsuragi are employed by Kao Corporation.

## ETHICAL REVIEW

The study was approved by the ethics committee of the Bokushinkai Medical Corporation Shirogane EXE Clinic (approval number: 2013‐0501; approval date: 8 May 2013).

## INFORMED CONSENT

Written informed consent was obtained from all study participants.
